# A nomogram based on A-to-I RNA editing predicting overall survival of patients with lung squamous carcinoma

**DOI:** 10.1186/s12885-022-09773-0

**Published:** 2022-06-29

**Authors:** Li Liu, Jun Liu, Xiaoliang Deng, Li Tu, Zhuxiang Zhao, Chenli Xie, Lei Yang

**Affiliations:** 1 The State Key Lab of Respiratory Disease, Institute of Public Health, Guangzhou Medical University, Xinzao, Panyu District, Guangzhou, 511436 China; 2 Department of Pulmonary and Critical Care Medicine, Guangzhou First People’s Hospital, the Second Affiliated Hospital of South China University of Technology, Guangzhou, 510080 China; 3 Department of Respiratory Medicine, Hospital of Changan, Dongguan, 523843 China; 4 Department of Respiratory Medicine, Fifth People’s Hospital of Dongguan, Dongguan, 523939 China

**Keywords:** Lung squamous cell carcinoma, A-to-I RNA editing, Overall survival, Nomogram

## Abstract

**Background:**

Adenosine-to-inosine RNA editing (ATIRE) is characterized as non-mutational epigenetic reprogramming hallmark of cancer, while little is known about its predictive role in cancer survival.

**Methods:**

To explore survival-related ATIRE events in lung squamous cell carcinoma (LUSC), ATIRE profile, gene expression data, and corresponding clinical information of LUSC patients were downloaded from the TCGA database. Patients were randomly divided into a training (*n* = 134) and validation cohort (*n* = 94). Cox proportional hazards regression followed by least absolute shrinkage and selection operator algorithm were performed to identify survival-related ATIRE sites and to generate ATIRE risk score. Then a nomogram was constructed to predict overall survival (OS) of LUSC patients. The correlation of ATIRE level and host gene expression and ATIREs’ effect on transcriptome expression were analyzed.

**Results:**

Seven ATIRE sites that were *TMEM120B* chr12:122215052A > I, *HMOX2* chr16:4533713A > I, *CALCOCO2* chr17:46941503A > I, *LONP2* chr16:48388244A > I, *ZNF440* chr19:11945758A > I, *CLCC1* chr1:109474650A > I, and *CHMP3* chr2:86754288A > I were identified to generate the risk score, of which high levers were significantly associated with worse OS and progression-free survival in both the training and validation sets. High risk-score was also associated with advanced T stages and worse clinical stages. The nomogram performed well in predicting OS probability of LUSC. Moreover, the editing of ATIRE sites exerted a significant association with expression of host genes and affected several cancer-related pathways.

**Conclusions:**

This is the first comprehensive study to analyze the role of ATIRE events in predicting LUSC survival. The AITRE-based model might serve as a novel tool for LUSC survival prediction.

**Supplementary Information:**

The online version contains supplementary material available at 10.1186/s12885-022-09773-0.

## Introduction

As the leading cause of cancer-related death, lung cancer has resulted in an estimated 1,796,144 deaths in 2020 [1]. Over the past two decades, individualized targeted therapy has been practiced in lung cancer with appreciable benefits in some patients [2]. However, there are still a substantial portion of patients who undergo non-response, side effects and adverse reactions after targeted therapy, underscoring the critical need for accurate prediction models for cancer prognosis and new therapeutic targets. Therefore, to identify underlying molecular alterations of cancer and to characterize prognostic molecular markers have important implications for personalized cancer treatment.

The leapfrog development of high-throughput sequencing technology and bioinformatics tool have deeply revealed the abnormally genetic and non-mutational epigenetic alternations in human genome and transcriptome for cancer, which are promising biomarkers as tools for cancer diagnosis, prognostic assessment, and therapy. Unlike gene expression [3–7], somatic mutation [8], genetic variant [9], and DNA methylation [10–13], all of which have been extensively explored for establishing cancer prognostic prediction model, no study exploit the RNA editing. RNA editing is a molecular process through which cells can make specific alterations in the chemical structure of RNA molecules after transcription. Over 70% of RNA editing in human is adenosine-to-inosine RNA editing (ATIRE), which convert adenosine to inosine [14]. Since inosine is recognized as guanosine by posttranscriptional regulatory machinery, this will cause potential recoding events in amino acid sequences, alternative splicing, and binding reprogramming of microRNAs or RNA binding proteins in untranslated region (UTR) [15, 16]. So far, several ATIRE events such as GABRA3 [17], AZIN1 [18], and miRNA-379-5p [19] editing have been reported to be associated with various cancer survival. Meanwhile, ATIRE affect cancer progression [20, 21], metastasis [22], tumorigenesis [23–25], and drug resistance [26]. These findings highlight potential application of ATIRE as cancer biomarker. Yet, no study establish cancer prognostic prediction model based on ATIRE and the performance is unknown.

Here, we aimed to develop a prediction model using ATIRE to predict overall survival (OS) of individuals affected by lung squamous cell carcinoma (LUSC). We identified OS-related ATIRE events by analyzing the whole ATIRE profiles and clinical data of LUSC from the Cancer Genome Atlas (TCGA) database, and constructed a nomogram for predicting LUSC OS based on ATIRE risk score and clinicopathological characteristics. We also evaluated the underlying mechanisms by which these ATIRE sites impact LUSC survival.

## Materials and methods

### Sample selection and data processing

**Fig. 1:**
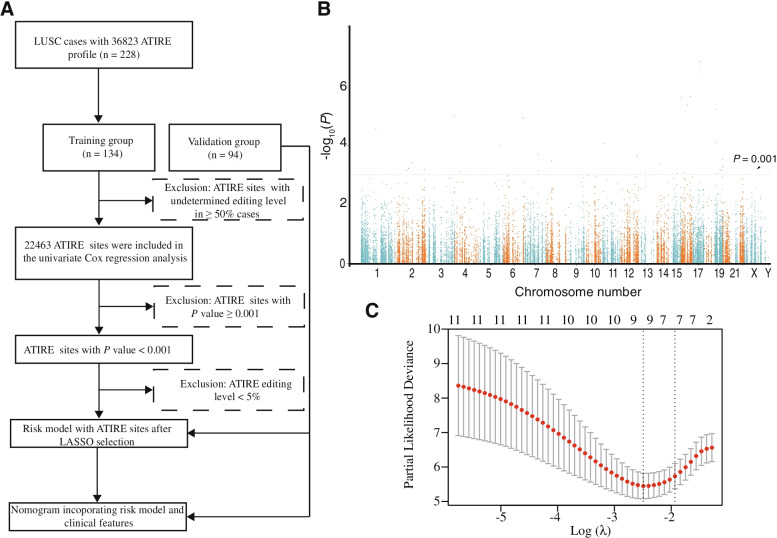
Identification of survival-related ATIRE sites in LUSC patients. **A** The workflow of survival-related ATIRE sites determination for LUSC and ATIRE-based nomogram construction. **B** A Manhattan plot depicts associations between all ATIRE sites and LUSC survival, taking the *P* values in –log10 scale from the univariate Cox-PH model as the X-axis, the chromosomal location of the ATIRE sites as the Y-axis. Dotted orange line indicates the cut off of significance with *P* value as 0.001. **C** Cross-validation for the selection of optimal ATIRE sites (lambda) and dotted vertical lines. ATIRE: A-to-I RNA editing

The ATIRE profiles of TCGA-LUSC samples were downloaded from the synapse website (https://www.synapse.org/#!Synapse:syn4382524) that was uploaded by Han L et al. [16]. The corresponding clinical information and gene expressional data were obtained from the TCGA database (https://portal.gdc.cancer.gov/). Only 228 samples who owned available ATIRE data were included in this study and randomly divided into a training set (*n* = 134) and a validation set (*n* = 94). A flowchart describing the data processing is provided in Fig. 1a. After removing the ATIRE sites with undetermined editing level in over 50% of cases, the univariate Cox proportional hazards (Cox-PH) regression was first used to explore OS-related ATIRE sites in the training set using the packages- “survival” and “survminer” in R (version 4.0.4), and the sites with *P* < 0.001 were considered to be significant. Then the least absolute shrinkage and selection operator (LASSO) algorithm was applied to determine the optimal prognostic ATIRE sites using the package-”glmnet” in R with penalty parameter tuning conducted by tenfold cross-validation [27]. Before LASSO, those sites with editing level less than 5% in over 90% of samples were removed, because extremely low level of editing is difficult to quantify precisely. These optimal ATIRE sites were used to generate a risk score with the coefficients from LASSO as follows:

$$\sum_{i}Coefficient\left({A-to-I site}_{i}\right)\times {editing level(A-to-I)}_{i}$$

### Development and validation of an ATIRE-based nomogram

The ATIRE risk score and clinicopathological features including T stage, N stage, age at diagnosis and gender were enrolled to establish an OS prognostic nomogram using the Cox-PH with the package- “rms” in R. The nomogram was based on proportionally converting each Cox regression coefficient in multivariate regression to a 0- to 100-point scale. Predictive performance of the nomogram was measured by the Harrell’s C-index and calibration with 100 bootstrap samples using the packages-”Hmisc”, “nomogramEx”, and “nomogramFormula” in R [28]. For validation, the total points of patients in the validation set were calculated according to the established nomogram, then Cox-PH regression was performed using the total point as a factor, and the Harrell’s C-index and calibration were assessed. Finally, a decision curve analysis were performed to determine the clinical usefulness of different prognostic models by quantifying the net benefits at different threshold probabilities using the package-”ggDCA” in R [29].

### Correlations between levels of ATIRE sites and expressions of target genes

To explore the possible mechanisms about how above ATIRE sites affect LUSC survival, correlations between the levels of them and their host genes’ expressions were analyzed in TCGA-LUSC tumor tissues using the Spearman rank test. Fragments Per Kilobase Million (FPKM) was used to demonstrate the expression value of target gene. Meanwhile, the correlation between the ATIRE risk score and ADAR1 expression were also analyzed.

### Effect of AITRE risk score on whole transcriptome expression and pathway

To further demonstrate the potential effect of ATIRE risk score on whole transcriptome expression, RNA-seq raw counts of TCGA-LUSC tumor tissues were downloaded and compared between the high- and low-risk patients using the package-”DESeq2″ in R. Then the Kyoto Encyclopedia of Genes and Genomes (KEGG) pathway and gene-set enrichment analysis (GSEA) were carried out using the package-”clusterProfiler” in R and GSEA 4.1.0 software, respectively. KEGG was permitted by Kanehisa Laboratories [30].

### Statistical analysis

For the aforementioned statistical tests in R software, the codes were presented in Supplementary methods. In addition, the effect of ATIRE risk score on OS as well as progression-free survival (DFS), demonstrated by hazard ratio (HR) and 95% confidence intervals (CI), was analyzed using the log-rank test, univariate or multivariate Cox-PH. Stratification analysis with regarding to clinicopathological features, and multiple interaction analysis between these factors and the risk score were performed using the Cox-PH. Normal-cancer difference in editing levels of ATIRE sites was tested by the student’s *t* test or unequal variances *t*-test. Editing levels among multiple groups were compared post hoc if the one way ANOVA was significant using the Dunnett’s multiple comparisons test. All tests were two-sided and evaluated by the Stata software (version 16.0). *P* < 0.05 was considered to be statistically significant.

## Results

### Baseline clinicopathological characteristics

The clinicopathological characteristics of TCGA-LUSC cases used in the current study are presented in Table 1. There was no significant difference in age, gender, smoking status, TNM stages and survival status between the training group and validation group. Moreover, increasing age (HR = 1.03, 95%CI = 1.00–1.10), male (HR = 1.68, 95%CI = 1.01–2.80), and advanced T stages (HR = 2.01, 95%CI = 1.05–3.90) were independent prognostic factors for LUSC OS (Supplementary Figure S1).

**Table 1:** Frequency distributions of demographic and clinicopathological features of LUSC cases

Variables	Training set (*n* = 134)	Validation set (*n* = 94)	*P* value
Age ( Mean ± SD, y)	68.3 ± 7.6	67.5 ± 9.0	0.445^a^
Gender			
Male	101 (73.4%)	67 (71.3%)	0.489^b^
Female	33 (24.6%)	27 (28.7%)	
Smoking status			
Yes	124 (92.5%)	89 (94.5%)	0.520^b^
No	10 (7.5%)	5 (5.3%)	
T stages			
1	33 (24.6%)	18 (19.1%)	0.324^b^
2	84 (62.7%)	58 (61.8%)	
3 + 4	17 (12.7%)	18 (19.1%)	
N stages			
0	91 (67.9%)	57 (60.6%)	0.515^b^
1	30 (22.4%)	25 (26.6%)	
2 + 3	13 (9.7%)	12 (12.8%)	
M stages			
0	133 (99.2%)	91 (96.8%)	0.166^b^
1	1 (0.8%)	3 (3.2%)	
Clinical stages			
I	72 (53.7%)	48 (51.1%)	0.248^b^
II	38 (28.4%)	21 (22.3%)	
III + IV	24 (17.9%)	25 (26.6%)	
Survival status			
Dead	53 (39.6%)	39 (41.5%)	0.678^c^
Alive	81 (60.4%)	55 (58.5%)	

^a^*P* value calculated by the student’s *t* test

^b^*P* value calculated by the two-side χ^2^ test

^c^*P* value calculated by the log-rank test

### Generation of ATIRE risk score for LUSC survival

A total of thirty-two ATIRE sites were identified to be associated with LUSC OS by the univariate Cox-PH analysis in the training set (*P* < 0.001; Fig. 1b). Among them, seven sites that are chr12:122215052A > I of *TMEM120B*, chr16:4533713A > I of *HMOX2*, chr17:46941503A > I of *CALCOCO2*, chr16:48388244A > I of *LONP2*, chr19:11945758A > I of *ZNF440*, chr1:109474650A > I of *CLCC1*, and chr2:86754288A > I of *CHMP3*, were selected as the optimal prognostic sites by the LASSO analysis and used to generate the ATIRE risk score (Fig. 1c). We named these sites according to their genomic locations on Human Feb. 2009 (GRCh37/hg19 Assembly). When editing levels of above ATIRE sites were grouped into low and high by the best cut-off point, determining by the X-Tile [31], all HRs of these sites are > 1 (Supplementary Figure S2), indicating that high editing of them were associated with unfavorable OS.

**Fig. 2:**
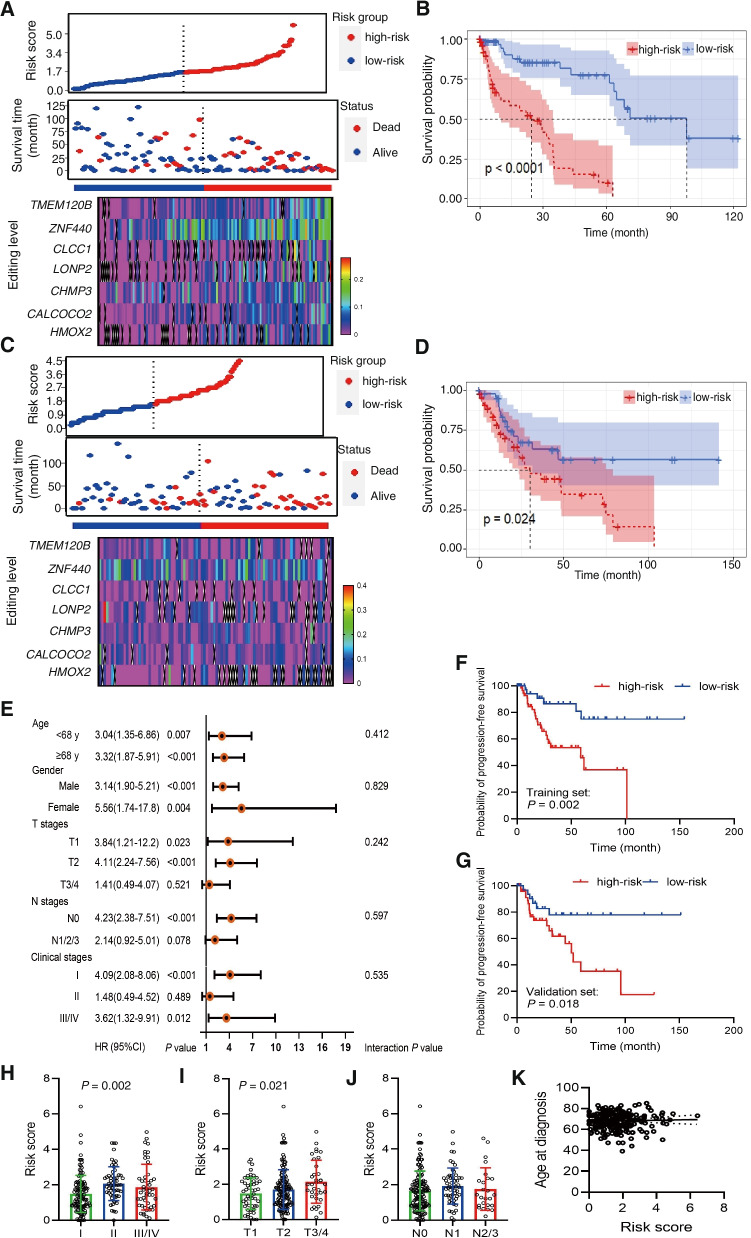
Associations between the ATIRE risk score and prognosis of LUSC patients. **A-D** Distribution of the ATIRE risk score, survival status, and editing levels of the 7 ATIRE sites in the training set (**A**) and validation set (**C**), and Kaplan–Meier plots to visualize the survival probabilities grouped by risk score in the training set (**B**) and validation set (**D**). *P* values were calculated by the log-rank test. **E** Stratification analysis of association between the ATIRE risk score and LUSC OS in different sub-groups with regarding to clinicopathological features. **F**, **G** Associations between the ATIRE risk score and progression-free survival in the training (**F**) and validation sets (**G**). *P* values were calculated by the log-rank test. **H-J** Differences of the ATIRE risk score among patients with different clinical stages (**H**), T stages (**I**), and N stages (**J**). *P* values were calculated by the one-way ANOVA test. **K** Correlation between the ATIRE risk score and age at diagnosis of LUSC patients. *P* value was calculated by the Spearman rank correlation test

The coefficients of each ATIRE site from the LASSO analysis were used to generate the ATIRE risk score as follows: (7.69 × *TMEM120B* chr12:122215052A > I) + (11.58 × *HMOX2* chr16:4533713A > I) + (4.19 × *CALCOCO2* chr17:46941503A > I) + (8.21 × *LONP2* chr16:48388244A > I) + (12.46 × *ZNF440* chr19:11945758A > I) + (2.63 × *CLCC1* chr1:109474650A > I) + (4.47 × *CHMP3* chr2:86754288A > I). Distribution of the risk scores, survival status, and editing levels of the 7 ATIRE sites are shown in Fig. 2A-D. Taking the median risk score as the cut-off point, those patients with high-risk score exerted significantly shorter median survival time and reduced probability of OS when compared to those with low-risk score in both the training (*P* < 0.0001) and validation cohorts (*P* = 0.024). However, we did not observed any significant interactions between the clinicopathological features and the risk score on affecting LUSC OS (Fig. 2E).

A significant association between the ATIRE risk score and progression-free survival (PFS) was also observed in both the training set (*P* = 0.002; Fig. 2F) and validation set (*P* = 0.018; Fig. 2G). In addition, high risk score was significantly associated with worse clinical stages (*P* = 0.002, Fig. 2H) and advanced T stages (*P* = 0.021, Fig. 2I), but not N stages (*P* = 0.297, Fig. 2J). Meanwhile, there was no significant correlation between the risk score and age at diagnosis (Fig. 2K).

### Establishment of ATIRE-based nomogram and predictive performance evaluation

**Fig. 3:**
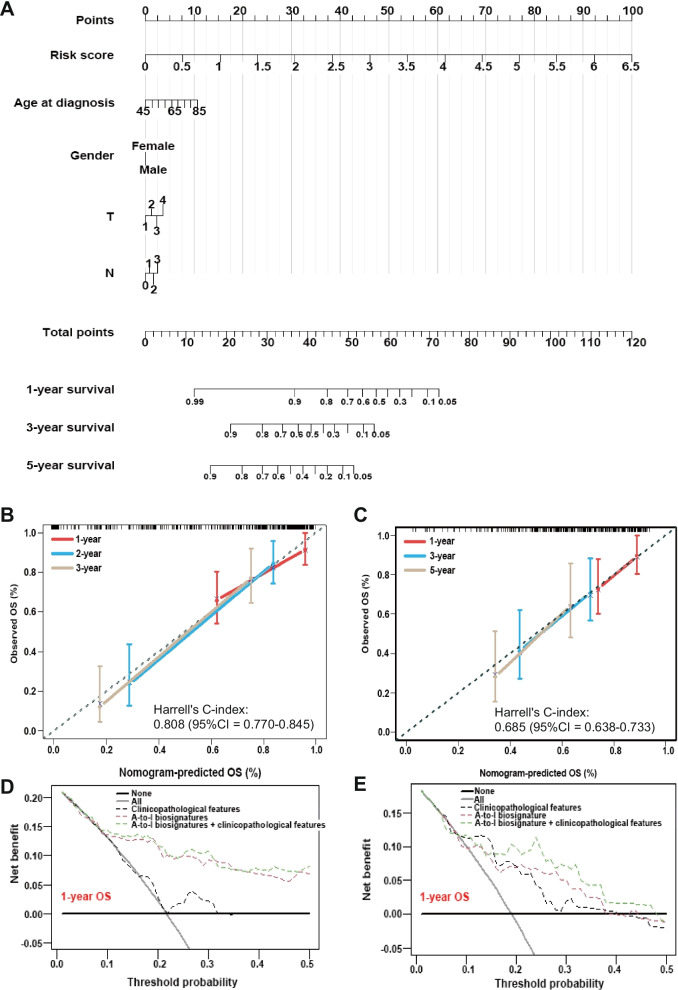
Performance of prognostic nomogram based on the ATIRE risk score and clinicopathological features. **A** The nomogram for predicting probabilities of 1-, 3- and 5-year OS in patients with LUSC; **B-C** Calibration curves show the agreement between the observed OS rate and nomogram-predicted OS rate at 1-, 3-, and 5-year in the training group (**B**) and validation group (**C**). **D-E** Decision curves depict the comparison in net benefits for predicting 1-year OS rate of different nomograms that are consistent of simple ATIRE risk score, clinicopathological features, and combination of ATIRE risk score and clinicopathological features in the training group (**D**) and validation group (**E**)

The nomogram was established with the ATIRE risk score and clinicopathological features including T stage, N stage, gender, and age at diagnosis (Fig. 3A). The calibration plots presented a superior agreement in both the training and validation sets between the observed OS rate and nomogram-predicted OS rate at 1-, 3-, and 5-year (Fig. 3B, C). The Harrell’s C-indexes were 0.808 (95%CI = 0.770–0.845) in the training set and 0.685 (95%CI = 0.638–0.733) in the validation set. Consistently, the decision curve showed that the ATIRE and clinicopathological features nomogram displayed a higher net benefit than the single ATIRE nomogram or clinicopathological features nomogram in both the training set and validation set on predicting 1- and 3-year OS rates (Fig. 3D, E; Supplementary Figure S3).

### ATIRE risk score is significantly associated with ADAR1 expression

Since ATIRE is majorly mediated by ADAR1 [32], we wondered whether adding ADAR1 level into the ATIRE nomogram would improve its performance. However, although there was a significant correlation between the ATIRE risk score and ADAR1 expression in TCGA-LUSC tumor tissues (Supplementary Figure S4), ADAR1 did not improve the performance of the established nomogram with similar Harrell’s C-indexes in both the training (i.e., 0.810) and validation (i.e., 0.665) sets. Moreover, we also constructed a nomogram integrating clinicopathological features and ADAR1. However, its performance was not as well as the ATIRE model with the Harrell’s C-indexes being 0.592 and 0.673 in the training and validation sets.

### Correlations between editing levels of ATIRE sites and expressions of host genes

**Fig. 4:**
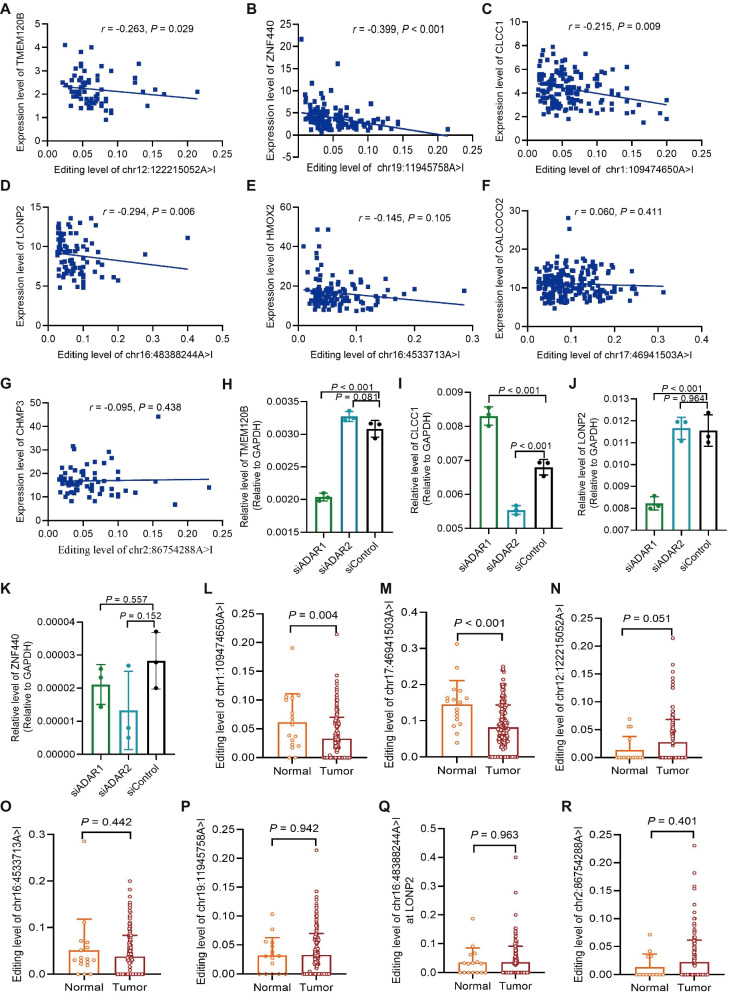
Effect of selected ATIRE sites on host genes’ expression. **A-G** Correlations between editing levels of the ATIRE sites and expressions of host genes in LUSC tumor tissues. FPKM value were used to demonstrate the expression of each gene. *P* and *r* were calculated by the Spearman correlation test. **H–K** Expression changes of indicated genes in response to knock-down of ADAR1/2. siADAR1, siADAR2, and siControl refer to siRNA targeting ADAR1, ADAR2 and a scramble siRNA, respectively. *P* value was calculated by the Dunnett’s multiple comparisons test. **L-R** Differences in editing levels of selected ATIRE sites between LUSC tumor tissues and normal tissues. *P* value was calculated by the student’s *t*-test or unequal variances *t*-test

ATIRE majorly regulate physiological and pathological processes via affecting host gene expression [33, 34]. As shown in Fig. 4A-D, there were significantly negative correlations between the chr12:122215052A > I and TMEM120B (*r* = -0.263, *P* = 0.029), the chr19:11945758A > I and ZNF440 (*r* = -0.399, *P* < 0.001), the chr1:109474650A > I and CLCC1 (*r* = -0.215, *P* = 0.009), and the chr16:48388244A > I and LONP2 (*r* = -0.294, *P* = 0.006). However, no significant correlation (Fig. 4E-G) was observed for the chr16:4533713A > I and HMOX2 (*P* = 0.105), the chr17:46941503A > I and CALCOCO2 (*P* = 0.411), and the chr2:86754288A > I and CHMP3 (*P* = 0.438). Furthermore, ADAR1 knock-down significantly altered the expressions of TMEM120B, CLCC1, LONP2, but not ZNF440 in A549 cells as the GEO data (accession number: GSE147487) shown (Fig. 4H-K). Moreover, knock-down of ADAR2 only changed the expression of CLCC1. In addition, the editing levels of CLCC1 chr1:109474650A > I (*P* = 0.004; Fig. 4L) and CALCOCO2 chr17:46941503A > I (*P* < 0.001; Fig. 4M) significantly differed between the tumor tissues and normal tissues. However, due to the limited sample size of normal tissues (*n* = 17), the difference of TMEM120B chr12:122,215,052 between the two group only displayed a clear trend to be significance (*P* = 0.051; Fig. 4N). However, no remarkable difference were observed for the other four sites (Fig. 4O-R).

### Differentially expressed genes and relevant biological pathways associated to the ATIRE risk score

**Fig. 5:**
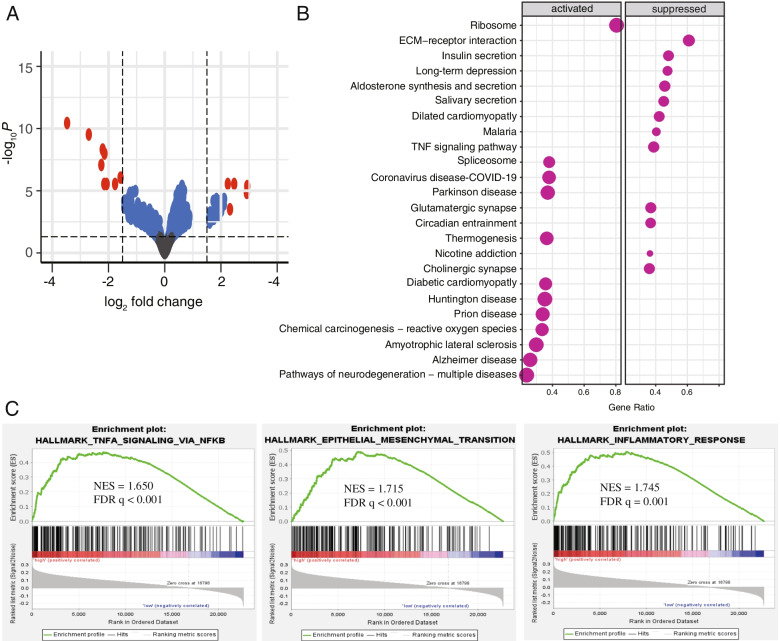
Differentially expressed genes and relevant biological pathways associated to the ATIRE risk score. **A** Visualization of differentially expressed genes with volcano plot in high-risk patients versus low-risk patients. The expression difference with a log twofold change of 1.5 (outer light gray broken vertical lines) and an adjusted *P* value of 0.05 (dark broken horizontal line) were labeled. Y axis refers to *P* values in–log10 scale, X axis refers to the fold change in log 2 scale. **B** Plot of the KEGG pathway enrichment analysis for ATIRE-related genes based on TCGA data. Y-axis represents pathways; X-axis represents the amount of the ATIRE-related genes enriched in KEGG pathways. The color and size of each bubble represent enrichment significance and the number of ATIRE-related genes enriched in KEGG. **C** GSEA enrichment plot of hallmarks in high-risk patients versus low-risk patients. KEGG: Kyoto Encyclopedia of Genes and Genomes; GSEA: Gene Set Enrichment Analysis; NES: normalized enrichment score. FDR: false discovery rate

Testing the gene expression difference among patients with high-risk and low-risk score revealed a total of 14 genes with significant differences between the two groups (Fig. 5A), such as SEZ6L [35], IGFBP1 [36], which are well-established to be implicated in lung cancer development. Further KEGG pathways analysis showed these genes were enriched in pathways involving extracellular matrix (ECM)-receptor interaction, tumor necrosis factor (TNF) signaling, nicotine addiction, chemical carcinogenesis-reactive oxygen species, and others (Fig. 5B). GSEA with the 50 hallmarks consistently found 21 that were significantly enriched (false discovery rate q < 0.05), such as TNF signaling via nuclear factor kappa B (NF-κB), epithelial mesenchymal transition (EMT), and inflammation response (Fig. 5C).

## Discussion

Establishment of cancer prognostic prediction model is not only critical to predict the prognosis outcome of cancer but also the selection of optimizing treatment. Progress in lung cancer OS predictive models, while substantial, was not as good as initially expected. Therefore, exploring new model, especially that based on novel molecular markers, is still of research value and practical significance with purpose to improve the availability of predictive model. Our study successfully identified seven ATIRE sites to generate an ATIRE risk score for LUSC risk stratification with regarding to prognosis, which was associated with LUSC OS, PFS, T stage, and clinical stage. The nomogram integrating the risk score and clinicopathological features exerts a well predictive performance on LUSC OS. To the best of our knowledge, this is the first study using ATIRE events as prognostic factors for predicting cancer survival.

ATIRE is increasingly being used to characterize cancer recently. Here, through Cox-PH regression and LASSO algorithm, seven OS-related ATIRE sites were identified to be optimal prognostic factor for LUSC. Most of these sites are located in genes that have been well-established to be implicated in lung cancer development except TMEM120B and ZNF440. For example, HMOX2 and CLCC1 have been characterized as biomarkers of tumor initiating cells, suppression of which will significantly increase cancer survival [37, 38]. CALCOCO2 is an autophagy receptor that contributes to autophagy addiction in Ras-driven lung cancer [39, 40]. CHMP3 is a tumor suppressor with lost expression across a wide range of human cancers and its high level predicts a favorite survival outcome of breast cancer patients [41]. Moreover, LONP2 promotes cervical carcinogenesis via oxidative stress [42]. These evidences supported functional underpinnings of association between these ATIRE sites and LUSC prognosis.

The underlying mechanisms about how does these sites relate to LUSC survival are still completely unknown. As reported, ATIRE may lead to non-synonymous amino acid mutations, mis-regulation of alternative splicing, disturbance codon preference, and microRNA-mRNA redirection or RNA-binding protein-mRNA redirection, thereby influencing gene expression or function [43]. Interestingly, significantly negative correlations between chr12:122215052A > I level and TMEM120B expression, chr19:11945758A > I level and ZNF440 expression, chr1:109474650A > I level and CLCC1 expression, chr16:48388244A > I level and LONP2 expression, were observed in LUSC tumor tissues. Meanwhile, knock-down of ADAR1, which is the key editing enzyme mediating ATIRE, caused significantly changed in the mRNA expressions of TMEM120B, CLCC1 and LONP2, indicating a post-transcriptional role of these sites on expressions of host genes. Since the four sites are all located in the 3’-UTR, it is highly plausible that they affect host genes’ expression via disturbing binding abilities of microRNAs or RNA-binding proteins. However, the mechanism has yet to be confirmed. Moreover, the chr17:46941503A > I is also located in the 3’-UTR of CALCOCO2 and chr16:4533713A > I is in the 5’-UTR of HMOX2. Considering mRNA levels may not be a good proxy for protein level for genes that undergo post-transcriptional regulation, the non-significant correlations between chr17:46941503A > I level and CALCOCO2 mRNA expression, and chr16:4533713A > I level and HMOX2 mRNA expression, don't necessarily mean that the editing of the two sites exert no effect on expression of host genes. Further analysis at the protein level is warranted. As an intron locus, chr2:86754288A > I is edited means it can be transcribed to pre-mRNA, endowing possible mechanism of the site beyond expression regulation such as mis-regulation of alternative splicing. Since biological rationality is one of the most important evidence supporting the causal relationship between molecular markers and disease development, further studies are warranted to elucidate the mechanisms underlying the associations between the seven ATIRE sites and LUSC survival.

Furthermore, significant differences in editing levels of chr17:46941503A > I of CALCOCO2, and chr1:109474650A > I of CLCC1 were observed between LUSC tumor tissues and normal tissues, indicating possible roles of these sites involving LUSC occurrence.

The ATIRE risk score derived from the aforementioned seven ATIRE sites, age at diagnosis, gender, T stage, and N stage were used to establish the nomogram. Generally, the nomogram exerted a medium accuracy on predicting OS of LUSC, displaying a better overall net benefit than the T, N stating system for predicting 1- and 3-year OS rate. Although in terms of validity, this ATIRE-based nomogram did not display a superior performance compared to previously published gene-expression-based nomograms as shown by the Harrell’s C-indexes, given an amount of gene-expression-based nomograms exerted Harrell’s C-indexes ranging from 0.65–0.85 [44–49]. However, in terms of determination reliability, quantification of AITRE level is more stable and reliable than that of gene expression. Compared to ATIRE test, gene expression determination is more easily affected by the RNA quality and PCR reaction, which may induce inter- and intra-individual variation. In addition, although the expression of ADAR1 was significantly correlated with the ATIRE risk score, the nomogram including ADAR1 and clinicopathological features did not perform as well as the ATIRE model. It is not surprising because ATIRE level is not absolutely determined by ADAR1, and the association strength of ADAR1 with LUSC survival is greatly inferior to the selected ATIRE sites, which were emerged as optimal survival-related sites from thousands of candidates. Meanwhile, the ADAR model was more susceptible to intra-individual variation. Adding ADAR1 also did not improve the performance of ATIRE-based nomogram, possibly due to the collinearity of ADAR1 expression and ATIRE risk score.

The current study has several limitations. First, since we only analyzed the TCGA data and lacked an external group to validate this ATIRE-based model, the reliability of its performance has a high risk of bias. Second, therapeutic schedule is important to show the application of prognostic nomogram for making individualized intervention strategies, but the information is unavailable in the TCGA database. Finally, there were selection and information bias in the process of subject recruitment and data analysis.

In conclusion, we for the first time generated an ATIRE risk score that are associated with OS, PFS, T and clinical stage of LUSC patients. The nomogram incorporating the ATIRE risk score and clinicopathological features exerted well predictive performance for LUSC OS. Large prospective sets are warranted to validate the robustness of this model to assess the application value in “real-world” clinic.

## Supplementary Information

**Additional file 1: Figure S1.** Multivariate Cox regression analyses of clinicopathological features on OS of LUSC patients.

**Additional file 2: Figure S2.** Kaplan-Meier plots to visualize survival probabilities for the 7 ATIRE sites in the training set. HRs were calculated by the Cox regression model.

**Additional file 3: Figure S3.** Decision curves depicting the comparison in net benefits for predicting 3-year and 5-year OS rate of different nomograms that are consistent of sole ATIRE risk score, sole clinicopathological features, and combination of ATIRE risk score and clinicopathological features in the training group and validation group.

**Additional file 4: Figure S4.** Correlation between the ATIRE risk score and ADAR1 expression.

**Additional file 5.** R codes used in the study.

## Data Availability

ALL data were downloaded from the synapse website (https://www.synapse.org/#!Synapse:syn4382524), and the corresponding clinical information from the TCGA database (https://portal.gdc.cancer.gov/). All these data are publicly available.
