# Supplementary figures and images for: A nomogram based on A-to-I RNA editing predicting overall survival of patients with lung squamous carcinoma
Source: BMC Cancer. 2022 Jun 29;22:715. doi: 10.1186/s12885-022-09773-0 (PMC9241197; doi:10.1186/s12885-022-09773-0)

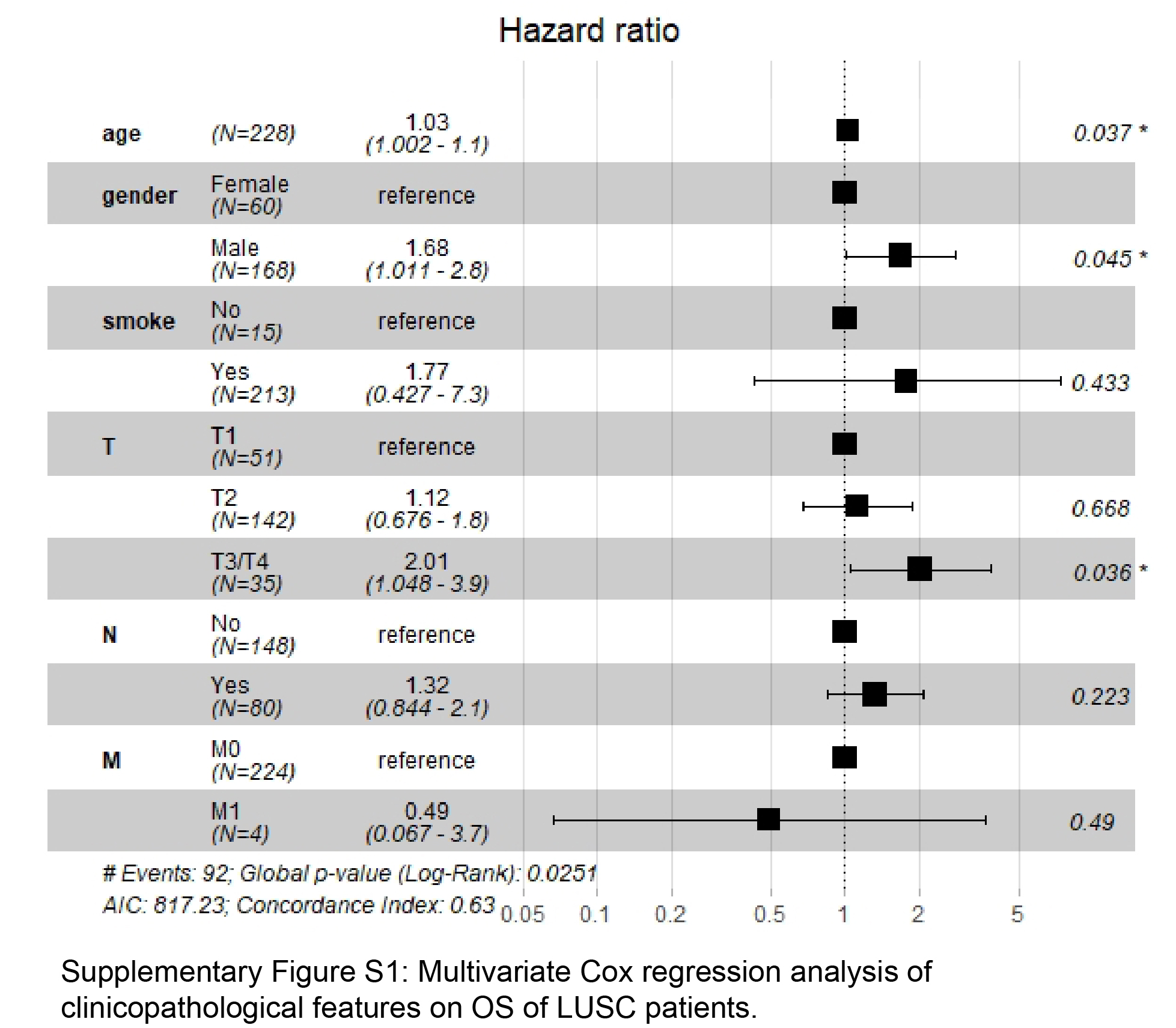

Supplement: Supplementary file 1 — Additional file 1: Figure S1. Multivariate Cox regression analyses of clinicopathological features on OS of LUSC patients. [file 12885_2022_9773_MOESM1_ESM.jpg]

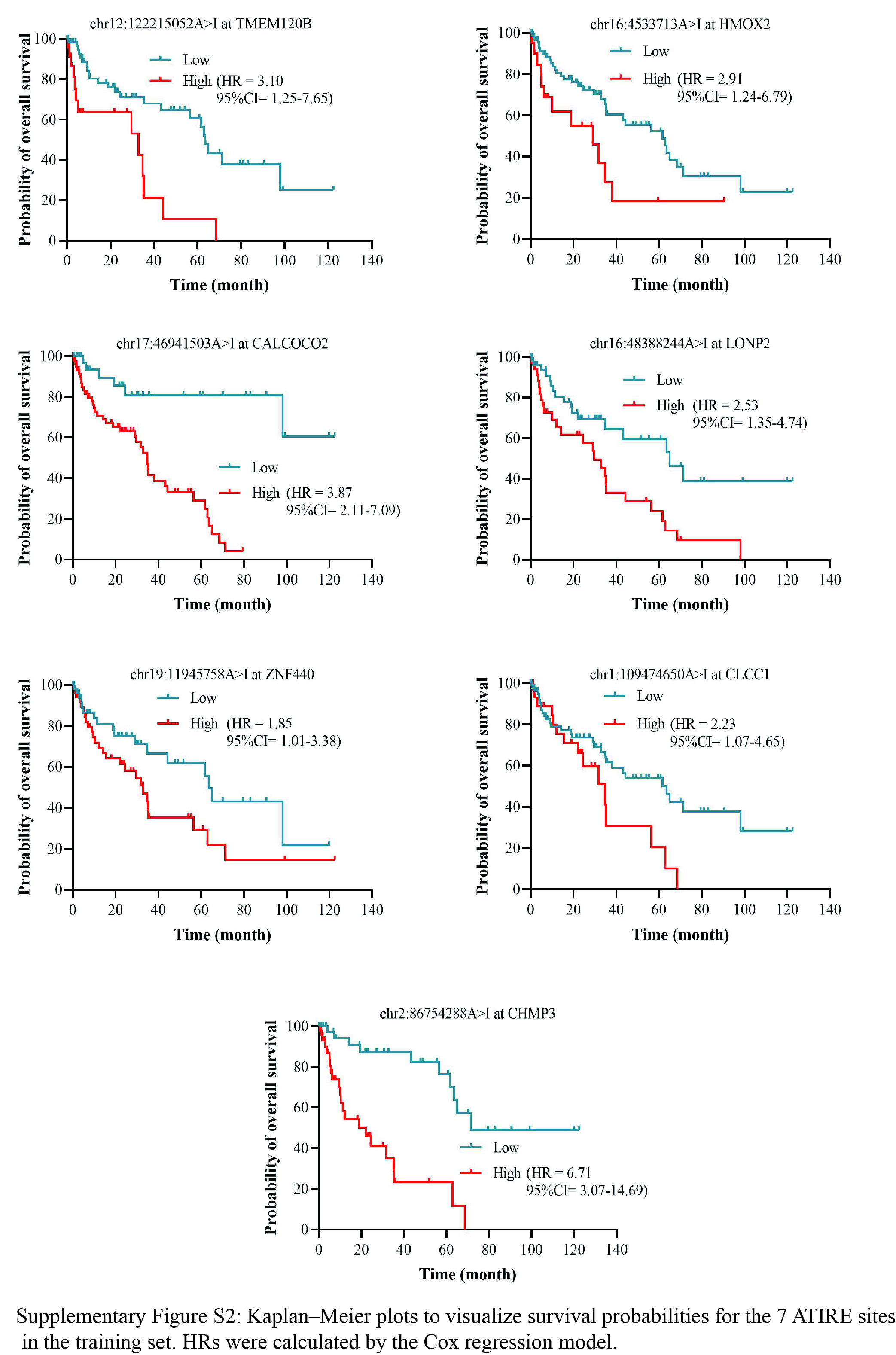

Supplement: Supplementary file 2 — Additional file 2: Figure S2. Kaplan-Meier plots to visualize survival probabilities for the 7 ATIRE sites in the training set. HRs were calculated by the Cox regression model. [file 12885_2022_9773_MOESM2_ESM.jpg]

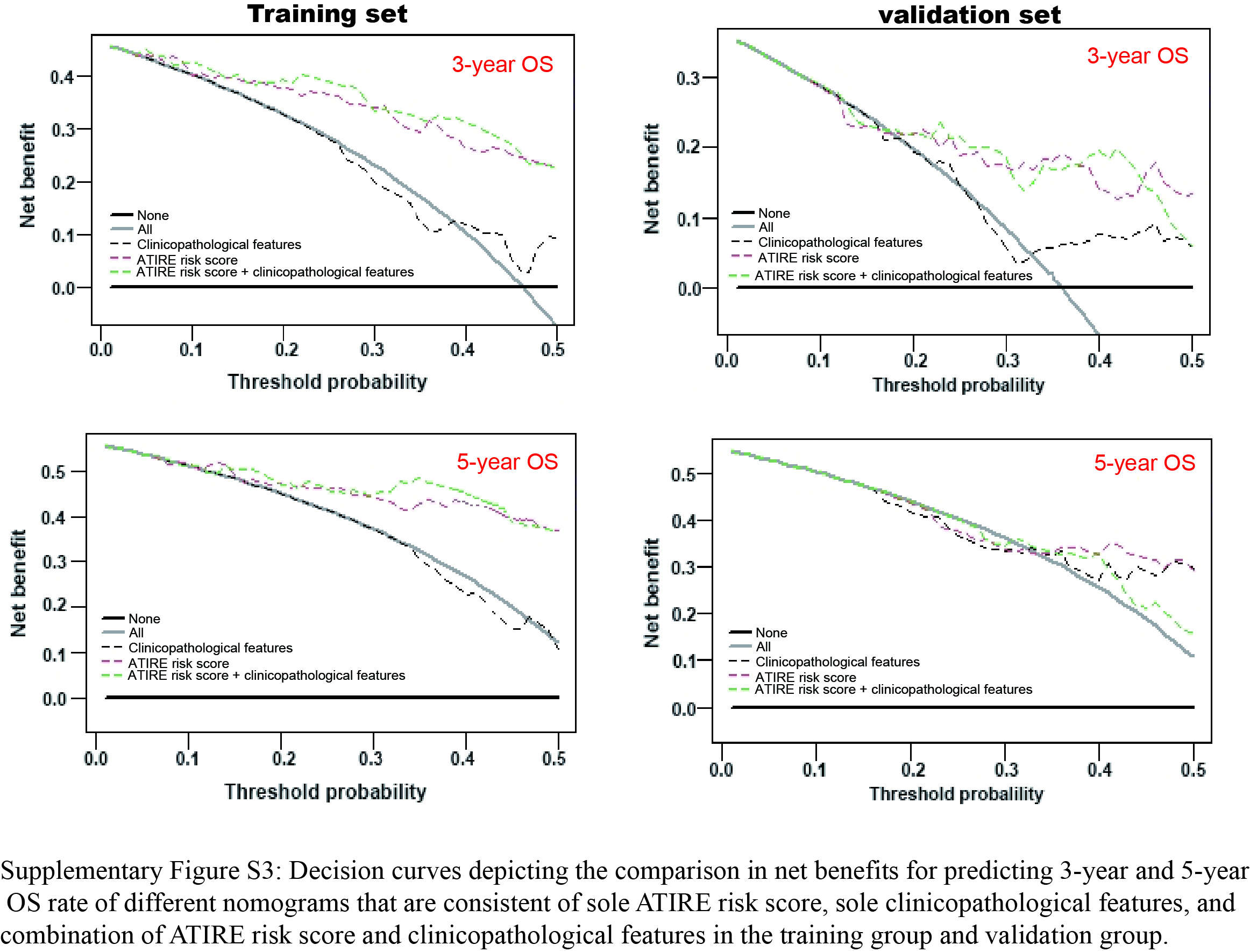

Supplement: Supplementary file 3 — Additional file 3: Figure S3. Decision curves depicting the comparison in net benefits for predicting 3-year and 5-year OS rate of different nomograms that are consistent of sole ATIRE risk score, sole clinicopathological features, and combination of ATIRE risk score and clinicopathological features in the training group and validation group. [file 12885_2022_9773_MOESM3_ESM.jpg]

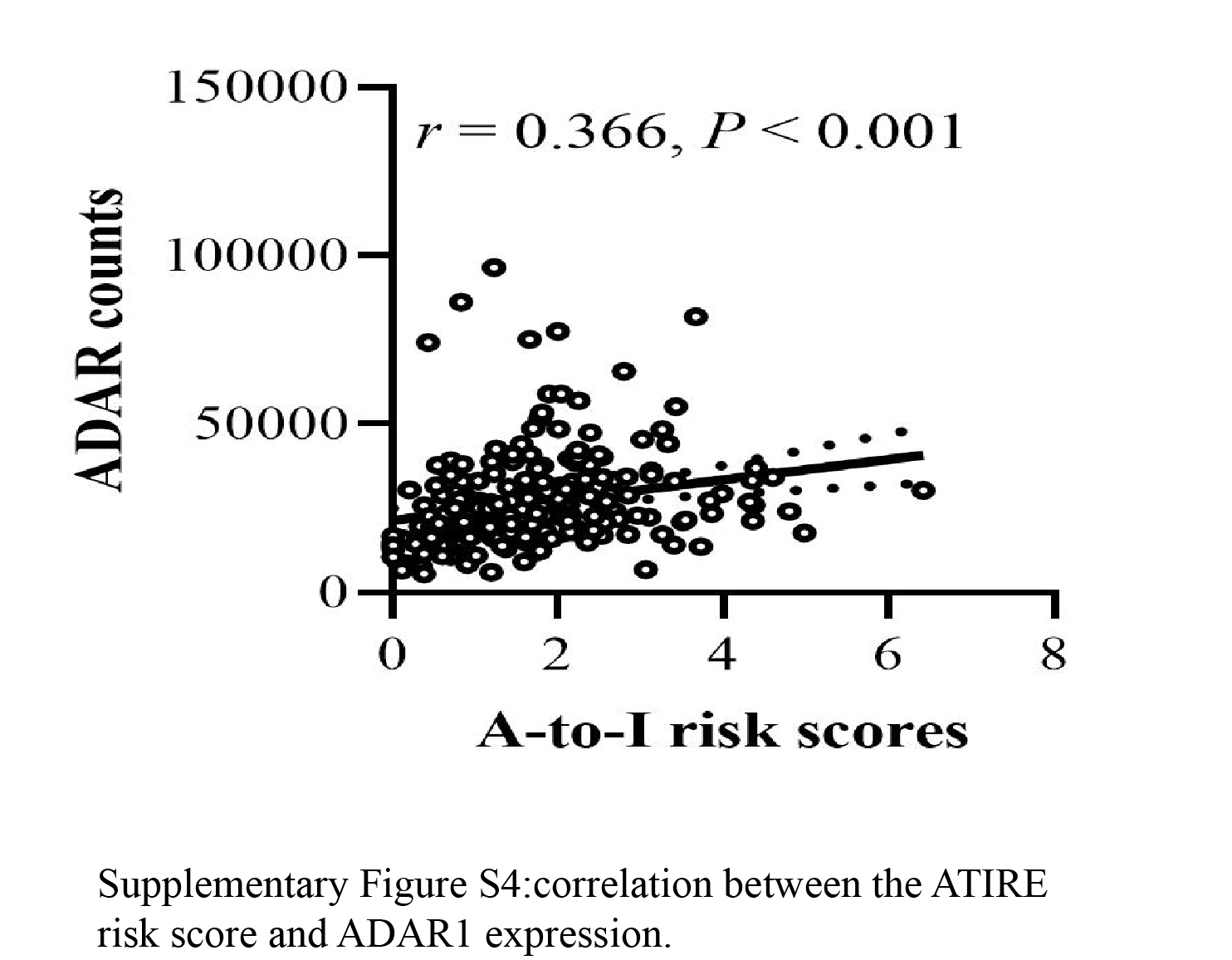

Supplement: Supplementary file 4 — Additional file 4: Figure S4. Correlation between the ATIRE risk score and ADAR1 expression. [file 12885_2022_9773_MOESM4_ESM.jpg]
