# Supplementary material for: A nomogram based on A-to-I RNA editing predicting overall survival of patients with lung squamous carcinoma
Source: BMC Cancer. 2022 Jun 29;22:715. doi: 10.1186/s12885-022-09773-0 (PMC9241197; doi:10.1186/s12885-022-09773-0)
Supplement: Supplementary file 5 — Additional file 5. R codes used in the study. [file 12885_2022_9773_MOESM5_ESM.docx]

**Supplementary methods: R codes used in the study**

***1. Determination of the OS-related ATIRE sites in the training set***

*# Merge the TCGA clinical data and ATIRE profile by TCGA ID:*

rm(list=ls())

library (survival)

setwd("…") *# source the data folder*

clinic<-read.csv(file="LUSC_TCGA.csv",header=TRUE) *# input the clinical data of training set*

AtoI<-read.csv(file="TCGA1.csv",header=TRUE) *# input the ATIRE data*

AIC<- merge(clinic, AtoI, by="ID")

write.csv(AIC,file="ATIRE_TCGA.csv") *# export the merged data*

*# Perform Cox-PH analysis (variable name-status: vital status; ob_time: survival time):*

AIE<-read.csv(file="ATIRE_TCGA",header=TRUE)

AIsite = AIE1[,27:ncol(AIE)] *# The number 27 is the column number for the first AITRE site*

list<- which(colSums(is.na(AIsite))>70) *#To remove the ATIRE sites with undetermined level in over 50% TCGA-LUSC samples*

AIsite_full<-AIsite[,-list]

p.val1 = c()

AI = c()

for (i in 1:ncol(AIsite_full)){

print(i)

AI = AIsite_full[,i]

KMdata = data.frame(status=AIE1$status, time = AIE1$ob_time, AIID=AI)

coxfit = coxph(Surv(time, as.numeric(status))~AIID, KMdata)

p.val1[i] = summary(coxfit)$coefficients["AIID","Pr(>|z|)"]

}

cox_results = data.frame(AIName = colnames(AIsite_full), pvalue = p.val1)

write.csv(cox_results, file="results.csv") *# export the results of P values from the Cox-PH analysis*

***2. Selection of optimal OS-related ATIRE sites by LASSO in the training set***

*# Before LASSO analysis, we manually constructed the dataset that only included samples with completely survival time (vital status and follow-up time), and the ATIRE sites with P< 0.001 and with an editing level less than 5% in >90% of samples. Also, sites locating in intergenic region were removed. We named the dataset as “LASSO.csv”.*

*# Perform LASSO analysis*

rm(list=ls(all=TRUE))

library(glmnet)

library(survival)

setwd("C:/Users/leiya/OneDrive/desk/A-to-I study/LUSC") *# source the data folder*

data<-read.csv(file="LASSO.csv", header=TRUE) *# input the data*

list<- which(colSums(is.na(data))>=25)

AIsite_full<-data[,-list] *# To further remove ATIRE sites with undetermined level in over 50% TCGA-LUSC samples*

list1<-which(rowSums(is.na(AIsite_full))>=1)

AIsite_full1<-AIsite_full[-list1,] *# To remove samples with missing data at any of the included ATIRE sites*

write.csv(AIsite_full1, file="training.csv") *# export the data*

fit_sur=Surv(AIsite_full1$ob_time,AIsite_full1$status)

data.x <- as.matrix(AIsite_full1[5:ncol(AIsite_full1)]) *# The number 5 is the column number for the first AITRE site*

cvfit = cv.glmnet(data.x,fit_sur, nfold=10, family = "cox")

plot(cvfit) *# Plot of cross-validation for the selection of optimal ATIRE sites (lambda) and dotted vertical lines (Fig 1C in the paper)*

coef.min = coef(cvfit, s = "lambda.min")

coef.min *# Show the coefficient for each ATIRE site*

***3. Illustration of distribution of the risk scores, survival status, and editing levels of the 7 ATIRE sites***

*# Before this analysis, we manually constructed the dataset that only included samples with completely survival time (vital status and follow-up time), and LASSO-selected ATIRE sites. Here, we take the data from validation set as an example. It was named as “validation.csv”.*

rm(list=ls())

library(ggrisk)

library(rms)

library(survival)

library(survminer)

setwd("C:/Users/leiya/OneDrive/desk/A-to-I study/LUSC") *# source the data folder*

lusc<-read.csv(file="validation.csv", header=TRUE) *# input the data*

lusc3 = data.frame(status=lusc$status,time = lusc$ob_time, fenshu=lusc$riskscore) *# risk score was used as an example, if you need to show all ATIRE sites, and replace and input all*

ddist <- datadist(lusc3)

options(datadist = "ddist")

coxfit <- cph(Surv(time, status) ~fenshu,x=T, y=T, surv=T,

data=lusc3)

ggrisk(coxfit,new.data = lusc3, cutoff.value = 'median',cutoff.x = 145,cutoff.y = -0.8,

code.0 = 'Still alive', code.1 = 'Already dead',code.highrisk = 'High risk',

code.lowrisk = 'Low irsk',title.A.ylab = 'Risk score',title.B.ylab = 'Survival time(month)', title.A.legend = 'Risk group',title.B.legend = 'Status',title.C.legend = 'Expression')

***4. Nomogram construction, Harrell's C-indexes calculation and calibration curve plotting.***

*# Taking ADAR1 expression as an example*

rm(list=ls())

library(survival)

library(survminer)

library(rms)

library(foreign)

library(Hmisc)

library(nomogramFormula)

library(DynNom)

library(nomogramEx)

library(dplyr)

library(ggtext)

library(regplot)

setwd("C:/Users/leiya/OneDrive/desk/A-to-I study/LUSC") # source the data folder

lusc<-read.csv(file="training_OS.csv", header=TRUE) # input the data of training set

lusc1 <- lusc[,c(6,7,10,11,15,16,19)] # build a new dataset that only includes the variables used

KMdata=data.frame(ID=lusc1$ID,Age_at_diagnosis=lusc1$age,Tstage=lusc1$Tstage, gender=lusc1$gender, Nstage=lusc1$Nstage, status=lusc1$status, time=lusc1$ob_time, ADAR=lusc1$ADAR) # build a dataset for nomogram construction with ADAR1 expression as an example

ddist <- datadist(KMdata)

options(datadist = "ddist")

KMdata$Tstage <- factor(KMdata$Tstage,labels=c("1", "2", "3", "4"))

KMdata$Nstage <- factor(KMdata$Nstage,labels=c("0", "1", "2", "3"))

KMdata$gender <- factor(KMdata$gender,labels=c("1", "2"))

fit <- cph(Surv(time, status) ~ADAR+ Age_at_diagnosis + Tstage + Nstage + gender,x=T, y=T, surv=T, data=KMdata, time.inc=60) # perform Cox-PH analysis

surv <- Survival(fit) # set the survival analysis function

nom<- nomogram(fit, fun = list(function(x) surv(12, x),function(x) surv(36, x), function(x) surv(60, x)),lp=F,funlabel=c("1-year survival", "3-year survival","5-year survival"),

maxscale=100,fun.at=c(0.99, 0.9, 0.8, 0.7, 0.6, 0.5, 0.4, 0.3,0.2,0.1,0.05)) # construct the nomogram for 1-year survival (12 months), 3-year survival (36 months), and 5-year survival (60 months)

plot(nom, lplabel="Linear Predictor",

xfrac=.5,varname.label=TRUE, varname.label.sep="=", ia.space=.2,

tck=NA, tcl=-1, lmgp=0.5,

points.label='Points', total.points.label='Total Points',

total.sep.page=FALSE,

cap.labels=FALSE,cex.var = 1,cex.axis = 1.1,lwd=50,

label.every = 1,col.grid = gray(c(0.8, 0.95))) # plot the nomogram

nomogramEx(nomo=nom,np=3,digit = 9) # set the nomogram function to calculate point

results<-formula_rd(nomogram=nom)

KMdata$points<-points_cal(formula=results$formula,rd=KMdata) # export point for each subject

# calculate the Harrell's C-indexes for nomogram in training set

sum.surv <- summary(coxph(Surv(time, status) ~points, data = KMdata))

c_index_se <-sum.surv$concordance

c_index<-c_index_se[1]

c_index

c_index.ci_low=c_index-c_index_se[2]

c_index.ci_low

c_index.ci_high=c_index+c_index_se[2]

c_index.ci_high

# plot the calibration curve for nomogram in training set

fit1 <- cph(Surv(time, status) ~points,x=T, y=T, surv=T,

data=KMdata, time.inc=12)

cal1 <-calibrate(fit1, cmethod = 'KM', method = 'boot' , u = 12, m =27, B = 1000)

plot(cal,

lwd = 2,

lty = 1,

errbar.col = c("#FF4040"),

xlim = c(0,1),ylim= c(0,1),

xlab = "Nomogram-prediced OS (%)",ylab = "Observed OS (%)",

cex.lab=1.2, cex.axis=1, cex.main=1.2, cex.sub=0.6)

lines(cal[,c('mean.predicted',"KM")],

type = 'b',

lwd = 3,

pch = 16,

col = c("#FF4040"))

mtext("")

box(lwd = 3)

abline(0,1,lty = 3,

lwd = 3,

col = c("#224444"))

fit3 <- cph(Surv(time, status) ~points ,x=T, y=T, surv=T,

data=KMdata, time.inc=36)

cal3 <-calibrate(fit3, cmethod = 'KM', method = 'boot' , u = 36, m =27, B = 1000)

fit5 <- cph(Surv(time, status) ~points,x=T, y=T, surv=T,

data=KMdata, time.inc=60)

cal5 <-calibrate(fit5, cmethod = 'KM', method = 'boot' , u = 60, m =52, B = 1000)

par(new=TRUE)

plot(cal3,

lwd = 2,

lty = 1,

errbar.col = c("#00BFFF"),

bty = "l",

xlim = c(0,1),ylim= c(0,1),

xlab = "Nomogram-prediced OS (%)",ylab = "Observed OS (%)",

cex.lab=1.2, cex.axis=1, cex.main=1.2, cex.sub=0.6)

lines(cal3[,c('mean.predicted',"KM")],

type = 'b',

lwd = 3,

pch = 16,

col = c("#00BFFF"))

par(new=TRUE)

plot(cal5,

lwd = 2,

lty = 1,

errbar.col = c("#CDB79E"),

bty = "l",

xlim = c(0,1),ylim= c(0,1),

xlab = "Nomogram-prediced OS (%)",ylab = "Observed OS (%)",

cex.lab=1.2, cex.axis=1, cex.main=1.2, cex.sub=0.6)

lines(cal5[,c('mean.predicted',"KM")],

type = 'b',

lwd = 3,

pch = 16,

col = c("#CDB79E"))

mtext("")

legend("topleft",

legend = c("1-year","3-year","5-year"),

col =c("#FF4040","#00BFFF","#CDB79E"),

lwd = 5,

cex = 1.2,

bty = "n")

# for validation set, points for each subject can be calculated by:

KMdata2$points<-points_cal(formula = results$formula,rd=KMdata2)

# Then repeat the above R protocol for calibration curve and C-index.

***5. Transcriptome difference analysis and KEGG analysis***

*# Before this analysis, we downloaded the whole transcriptome data of TCGA-LUSC tumor tissues and matched it with the ATIRE risk score by TCGA ID. The data was named as “exp.csv”. All subjects were grouped based on the risk score.*

rm(list=ls())

library(DESeq2)

library(limma)

library(edgeR)

library(ggplot2)

library(ggthemes)

library(AnnotationDbi)

library(org.Hs.eg.db)

setwd("C:/Users/leiya/OneDrive/desk/A-to-I study/LUSC") # source the data folder

gsea<-read.csv("exp.csv",header=TRUE) # input the data of training set

expr_df <- gsea

metadata <- data.frame(sample_id = colnames(expr_df)[-1])

sample <- factor(c(rep("low",113), rep("high",100))) # Number of low risk samples is 113

metadata$sample <- relevel(factor(sample),"low")

dds <-DESeqDataSetFromMatrix(countData=expr_df,

colData=metadata,

design=~sample,

tidy=TRUE)

dds <- dds[rowSums(counts(dds))>1,]

dds <- DESeq(dds)

normalized_counts <- as.data.frame(counts(dds, normalized=TRUE))

contrast <- c("sample", "low", "high")

dd1 <- results(dds, contrast=contrast, alpha = 0.05)

plotMA(dd1, ylim=c(-2,2))

dd2 <- lfcShrink(dds, contrast=contrast, res=dd1,type="ashr")

plotMA(dd2, ylim=c(-5,5))

summary(dd2, alpha = 0.05)

library(dplyr)

library(tibble)

res <- dd2 %>%

data.frame() %>%

rownames_to_column("ID")

# Transfer the Ensemble gene ID into gene symbol

res$symbol <- mapIds(org.Hs.eg.db,

keys=res$ID,

column="SYMBOL",

keytype="ENSEMBL",

multiVals="first")

# Transfer the Ensemble gene ID into entrez ID for KEGG analysis

res$entrez <- mapIds(org.Hs.eg.db,

keys=res$ID,

column="ENTREZID",

keytype="ENSEMBL",

multiVals="first")

gene_df <- res %>%

dplyr::select(ID, symbol, entrez, log2FoldChange, pvalue, padj) %>% #

filter(entrez!="NA") %>%

distinct(entrez,.keep_all = T)

geneList <- gene_df$log2FoldChange

names(geneList) = gene_df$entrez

geneList = sort(geneList, decreasing = TRUE)

head(geneList)

write.csv(gene_df, 'results.csv', quote = F, row.names = T) # Export the results of transcriptome difference analysis

# Perform KEGG analysis

library(clusterProfiler)

gseaKEGG <- gseKEGG(geneList = geneList,

organism = 'hsa',

nPerm = 1000,

minGSSize = 20,

pvalueCutoff = 0.1,

verbose = FALSE)

library(ggplot2)

dotplot(gseaKEGG,showCategory=12,split=".sign")+facet_grid(~.sign)
